# The Effect of Donor Human Milk Fortification on The Adhesion of Probiotics In Vitro

**DOI:** 10.3390/nu12010182

**Published:** 2020-01-09

**Authors:** Anastasia Mantziari, Satu Tölkkö, Artur C. Ouwehand, Eliisa Löyttyniemi, Erika Isolauri, Seppo Salminen, Samuli Rautava

**Affiliations:** 1 Functional Foods Forum, Faculty of Medicine, University of Turku, Itäinen Pitkäkatu 4A, 20520 Turku, Finland; satu.tolkko@utu.fi (S.T.); seppo.salminen@utu.fi (S.S.); 2 DuPont Nutrition and Biosciences, Sokeritehtaantie 20, 02460 Kantvik, Finland; arthur.ouwehand@dupont.com; 3 Unit of Biostatistics, Department of Clinical Medicine University of Turku, Kiinamyllynkatu 10, 20520 Turku, Finland; eliisa.loyttyniemi@utu.fi; 4 Department of Pediatrics, University of Turku and Turku University Hospital, Kiinamyllynkatu 4-8, 20520 Turku, Finland; eriiso@utu.fi (E.I.); samulirautava@gmail.com (S.R.)

**Keywords:** human milk fortifier, infant nutrition, preterm infant, *Lactobacillus rhamnosus* GG, *Bifidobacterium animalis* subsp. *lactis* Bb12

## Abstract

Preterm delivery complications are the primary cause of death among children under the age of five. Preventive strategies include the use of pasteurized donor human milk (DHM), its fortification with human milk fortifiers (protein supplements), and supplementation with probiotics. Our aim was to examine the impact of DHM and fortified DHM (FDHM) on the mucus adhesion properties of two widely used probiotics. The study covered two forms of human milk fortifier, liquid and powdered, with or without probiotics and storage at 4 °C for 24 h. To test the adhesion properties of the probiotic strains, DHM+probiotics and FDHM+probiotics were prepared and added to immobilized mucus isolated from the stool of healthy Finnish infants. The probiotic adhesion was then measured by liquid scintillation. Our results suggest that addition of liquid or powdered human milk fortifier in donor human milk had no impact on probiotic adhesion. In addition, given the increased adhesion of probiotics suspended in buffer, other matrices should be further studied. These factors need to be considered when designing future intervention strategies using probiotics in preterm infants.

## 1. Introduction

In 2018 alone, an estimated 5.3 million children under the age of five have died, with preterm birth complications as the leading cause of these deaths (18%) [1]. For those infants who are receiving intensive care, the concerns are not limited to their immediate problems, but also extend to the long-term health issues that they might face. Research shows that premature infants have a higher risk of respiratory and digestive issues, sight and hearing problems, and learning difficulties [2].

Infant nutrition is a key variable in the infant mortality puzzle. Especially nutritional interventions designed for the preterm infant aim at achieving postnatal growth equal to the one the infant would have if it was still in-utero. Despite its strong benefits for preterm infants in Neonatal Intensive Care Units (NICUs), the mother’s own milk is not always available. In these cases, pasteurized donor human milk (DHM) is the preferred alternative feeding as it is also thought to contribute to a reduction in, among others, necrotizing enterocolitis (NEC) risk. Through its use, the hospital is minimizing the substantial costs that would be associated with the treatment of conditions associated with prematurity [3,4]. DHM is therefore regarded as cost-effective. However, human milk alone is not sufficient for optimal growth of the newborn, as the provision of nutrients, and more importantly protein, is lower than the quantities needed for the rapid postnatal growth [5]. Indeed, research suggests that premature infants fed with protein-fortified human milk (FDHM) had small but significant increases in weight gain rates compared with those fed DHM [6]. Human milk should be therefore fortified with nutritional supplements known as human milk fortifiers (HMFs), which provide this source of protein and macronutrients [7].

During the pasteurization process, DHM is stripped of the live bacteria that it naturally contains, and its supplementation with specific probiotics has been shown to significantly reduce mortality and improve feed tolerance in very preterm infants [8]. Probiotics are defined as “live micro-organisms that, when administered in adequate amounts, confer a health benefit on the host” [9]. Adhesion to the intestinal mucosa is considered one of the criteria for a microorganism suggested to be a probiotic. By adhering to intestinal mucus, probiotics may promote local host defenses and act against pathogens [10]. Another rationale for the inclusion of probiotics in preterm infant nutrition is the difference between the gut microbiota composition of preterm infants compared with that of term infants. This possibly contributes to the development of NEC [11]. Therefore, different microbiota modulation interventions, including probiotic therapy, are employed to establish a normal commensal gut microbiome and prevent the development of NEC. According to a meta-analysis of clinical trials, probiotic supplementation is associated with reduced risk of NEC and death in preterm neonates [12].

Despite the benefits of the above-mentioned interventions, there is limited knowledge on whether FDHM can serve as an optimal carrier for probiotics. Thus, the main objective of the current study was to investigate the effect of DHM and FDHM on the adhesion of two well-known and well-characterized probiotic strains and their combination using the human infant intestinal mucus model. In addition, we aimed to investigate how the type of fortifier influences probiotic adhesion when present in DHM. Bovine-derived HMFs exist in two forms, liquid and powdered, and both are used in the NICU. The powdered form is preferred to reduce the dilution of human milk, while the liquid is used when there is a limited quantity of human milk. We also assessed whether cold storage influences the probiotic adhesion capacity, as this is relevant for human milk banks that may refrigerate FDHM for up to 24 h.

## 2. Materials and Methods

### 2.1. Probiotic Strains and Culture Conditions

The probiotic strains tested for their adhesion ability were *Lactobacillus rhamnosus* GG (LGG) (Valio Ltd., Helsinki, Finland) and *Bifidobacterium animalis* subsp. *lactis* Bb12 (DSM15954) (Chr. Hansen, Copenhagen, Denmark). The probiotic strains were reactivated from stocks stored at −70 °C in 25% glycerol (1% inoculum) and were used as single strains or as a mixture.

To label the strains metabolically, 5 μL from each of the reactivated probiotics was inoculated with 10 μL of [3H]-thymidine (Perkin Elmer NET355001MC, PerkinElmer, Waltham, MA, USA) in 1 mL of the respective media. LGG was grown aerobically in de Man, Rogosa, and Sharpe (MRS; Merck, Darmstadt, Germany) broth at 37 °C for 18–20 h, while Bb12 was grown in Gifu Anaerobic Medium (GAM; Nissui Seiyaku Co., Tokyo, Japan) broth for 48 h at 37 °C under anaerobic conditions (10% H_2_, 10% CO_2_, and 80% N_2_; Concept 400 anaerobic chamber, Ruskinn Technology, Leeds, UK). The strains were harvested by centrifugation (2000× *g*, 5 min), washed twice, and resuspended in HEPES (*N*-2-hydroxyethylpiperazine-*N*′-2-ethanesulphonic acid)-Hanks buffer (HH; 10 mM of HEPES; pH 7.4). The optical density (OD_600nm_) was adjusted to 0.25 ± 0.01 (~10^8^ CFU/mL). In this study, we aimed to compare the probiotic adhesion between DHM and FDHM, considering DHM as control. To prepare DHM + probiotics and FDHM + probiotics, the same volume used to adjust the optical density was added to DHM and FDHM. To test if the adhesion of LGG is influenced by the presence of Bb12 and vice versa, radiolabeled LGG was mixed with an equal amount (~5 × 10^7^ CFU/mL) of unlabeled Bb12 and the other way around.

To confirm that the viability of the probiotics would be maintained, unlabeled stocks of LGG, Bb12, and their combination were prepared as specified above and added to powdered and liquid FDHM. Supplemented samples were kept in the fridge (+4 °C) for 24 h. Serial dilutions were prepared and spread onto MRS (for LGG and combination) and GAM (for Bb12 and combination) plates in triplicate and were incubated aerobically and anaerobically for 48 and 72 h at 37 °C, respectively. The viability experiment was performed three times. The microbial counts were expressed as log_10_CFU/mL, and a selection of LGG and Bb12-like colonies was confirmed by colony PCR with strain-specific primers. The primers used were respectively Lrhamn1 (5′-CAATCTGAATGAACAGTTGTC-3′), Lrhamn2 (5′-TATCTTGACCAAACTTGACG-3′), BB12-tuf-F (5′-GTGTCGAGCGCGGCAA-3′), and BB12-tuf-R (5′-CTCGCACTCATCCATCTGCTT-3′). In short, selected colonies were resuspended in 50 μL of RNAse-free water and incubated for 10 min at 95 °C. Tubes were fast-chilled in ice for 2 min. Debris was then precipitated by centrifugation at 13,300 rpm for 15 min, and the supernatant containing DNA was stored at −20 °C for later analysis. Amplification of the DNA was performed using a PCR iCycler apparatus (Bio-Rad, Espoo, Finland). Initially, PCR reactions were carried out using 1 μL (10 ng/μL) of DNA template, 12.5 μL of Kapa2G Robust HotStart ReadyMix (2X) (Kapa Biosystems, South Africa), 10.5 μL of RNAse free H_2_O, and 10 pmol/μL of each gene-specific primer in a final volume of 25 μL. PCR amplification conditions for LGG and Bb12 were respectively as follows: initial denaturation step at 95 °C for 3 min; 35 cycles of denaturation at 95 °C for 15 s, annealing at 50 °C for LGG, 60 °C for Bb12, extension at 72 °C for 15 s, followed by a final extension step at 72 °C for 5 min. PCR products were separated and confirmed by electrophoresis in a 1% (*w*/*v*) agarose gel and visualized with ethidium bromide under ultraviolet (UV) light.

### 2.2. Collection and Pasteurization of Human Milk

Frozen human milk was donated by five healthy mothers. Human milk from each mother was thawed overnight in a refrigerator and mixed by gently inverting the tubes. The milk from five different mothers was then pooled in equal parts under a laminar hood and Holder pasteurized (62.5 °C for 30 min) in a thermostatically controlled water bath (model GD100; Grant Instruments Ltd., Cambridgeshire, UK). In short, the Duran glass bottle containing the pooled DHM was submerged into a preheated water bath (63 °C) and then rapidly cooled (<4 °C). To make sure that the temperature of the pooled DHM was maintained at 62.5 °C for 30 min, we placed a temperature probe in a bottle containing Milli-Q water. After pasteurization, the DHM was divided into three aliquots of 50 mL and stored at −80 °C for later analysis. Before the adhesion experiment, DHM was thawed overnight in a refrigerator.

### 2.3. Human Intestinal Mucus Extracted from Infant Feces

The fecal samples were obtained from two age groups of healthy Finnish infants: 0–6-month-old infants that were exclusively breastfed (mucus < 6; *n* = 5) and from 6–12-month-old infants that were exclusively breastfed and introduced to solid food (mucus > 6; *n* = 5).

To extract intestinal mucus from infant feces, 2 g from each fecal sample was suspended at 4 °C in phosphate-buffered saline (PBS; pH 7.2; 10 mM phosphate) containing protease inhibitors and sodium azide. Fecal extracts were prepared by centrifuging the suspension at 13.800× *g* at 4 °C for 30 min. The crude mucus was then isolated by dual ethanol precipitation [13,14] and further purified by fractionation using an XK26 column packed with Sepharose CL-4B (GE Healthcare, Chicago, IL, USA). In short, 5 mL of the crude mucus was applied to the column and fractionated using a peristaltic pump (Alitea XV U1-M; Alitea, Stockholm, Sweden) at a 3.1% rpm flow rate. This procedure was repeated once more. The void volume was dialyzed against distilled water at 4 °C overnight and lyophilized. For each group, equal amounts of lyophilized mucus glycoproteins from each individual were pooled to make a stock suspension of 10 mg/mL in HH.

### 2.4. In Vitro Adhesion Assay

Mucus glycoproteins were diluted (0.5 mg/mL) in HH, and 100 μL/well of the suspension was immobilized on a polystyrene microtiter plate (Maxisorp, Nunc, Denmark) by overnight incubation at 4 °C. Excess mucus was removed by washing the wells twice with 200 μL of HH. Two forms of HMF, liquid and powdered (Enfamil^®^ Human Milk Fortifier Acidified Liquid and Enfamil^®^ Human Milk Fortifier Powder respectively; Mead Johnson, Evansville, IN, USA), were tested and diluted in DHM following the manufacturer’s instructions. DHM+probiotics and FDHM+probiotics were prepared and added to the immobilized mucus. To study whether probiotic adherence to mucus is increased after mucus treatment with FDHM, the wells were incubated at first only with FDHM for an hour at 37 °C (INC37). Before the addition of probiotics, all wells were washed twice with 200 μL of HH.

To quantify the adhesive ability of the probiotic strains, radioactively labeled bacteria in HH, DHM, or FDHM (100 μL) were added to the wells and incubated at 37 °C for 1.5 h. The wells were then washed three times with 200 μL of HH to remove any unattached bacteria. The adhered bacteria were released and lysed with 1% SDS in 0.1 M NaOH (200 μL per well) by incubation at 60 °C for 1 h. The lysate was then removed and mixed with the scintillation liquid (Ultima Gold™ XR; Perkin Elmer, Waltham, WA, USA). The radioactivity of the lysed suspension was measured with a 1450 Microbeta Liquid Scintillation Counter (Wallac Oy, Turku, Finland).

### 2.5. Statistical Analysis

#### 2.5.1. Adhesion Assay

The adhesion assay was carried out as three independent experiments, with each experiment performed in triplicate. The adhesion ratio (in %) was calculated by dividing the radioactivity of the probiotics bound (triplicate 100 μL samples) by the radioactivity of the probiotics added to the infant intestinal mucus. The effect of the probiotic combination, presence of DHM and type of HMF, and subject age on the adhesion rate were evaluated with a two-sample t-test. A paired t-test was used to evaluate the statistical significance of the differences in the ability of each probiotic to adhere to mucus after supplemented to FDHM and subjected to cold storage (4 °C) for 24 h.

#### 2.5.2. Viability Assay

The enumeration data were expressed as log_10_ CFU/mL. A paired t-test was conducted to examine any significant differences in the viable cell count (CFU/mL) between 0 h (baseline) and 24 h of storage at 4 °C for each probiotic and type of fortifier separately. Statistical significance was set at 0.05 (two-tailed). All statistical analyses were carried out using the IBM SPSS statistics 23.0 software (IBM Corp., Armonk, NY, USA).

## 3. Results

**Figure 1:**
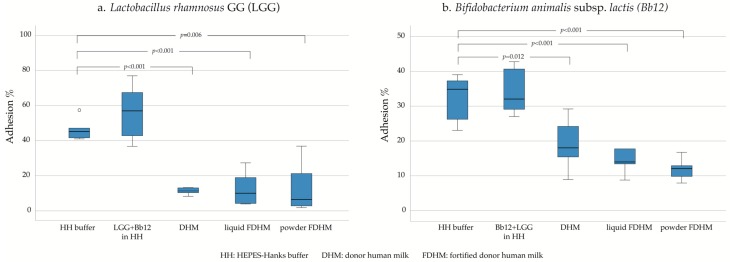
The adherence of *L. rhamnosus* GG (**a**) and *B. lactis* Bb12 (**b**) to human intestinal mucus glycoproteins in vitro as presented with box plot with outliers marked with a circle (o).

The percentage of adhesion varied among LGG and Bb12, but both tended to adhere in high numbers to the infant mucus. The adhesion of LGG in HH buffer, when assessed in combination with Bb12 (56.3%), was similar when compared with LGG assessed as a single strain (46.3%, *p* = 0.19; Figure 1a). The same was observed for Bb12 (combination with LGG: 34%, Bb12 alone: 32.5%, *p* = 0.72; Figure 1b). The presence of DHM, on the other hand, significantly reduced the adhesion capacity of both probiotics (LGG: 11.3%, *p* < 0.001; Bb12: 19.1%; *p* = 0.012). The type of fortifier did not play a role in adhesion efficacy of the tested probiotics but in general, its presence negatively affected the adhesion. More specifically, liquid FDHM (12.4%, *p* < 0.001) and powdered FDHM (13.8%, *p* = 0.006) appeared to suppress the adhesion of LGG similarly. The same was observed for Bb12 (liquid FDHM: 14.3%, *p* < 0.001; powdered FDHM: 11.9%; *p* < 0.001). Moreover, it was shown that specifically for Bb12, the second-best adhesion was achieved by supplementing probiotics after the mucus had been exposed to powder FDHM (21.5%) as compared with administering probiotics directly in powder FDHM (11.9%, *p* = 0.012; Supplementary Figure S1).

**Figure 2:**
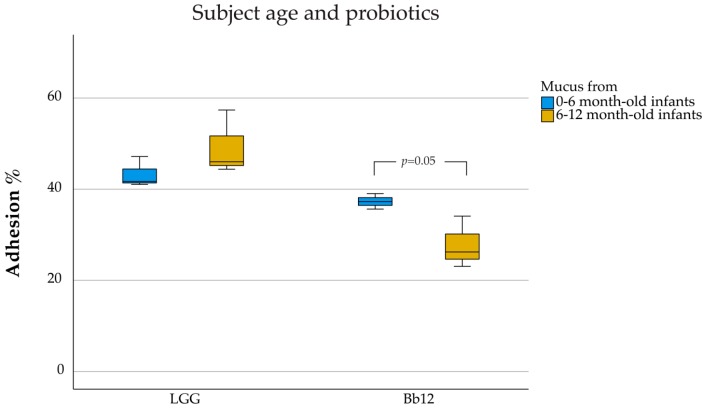
Adhesion of *L. rhamnosus* GG and *B. lactis* Bb12 as affected by age of mucus donor. Box-plot graphs represent the median and quartiles of adhesion (%) for the three independent experiments in triplicate. Outliers are marked with a circle (o).

Subject age was an important factor when determining the adhesion of probiotics alone to mucus, depending on the strain used. Mucus isolated from >6-month-old infants (49.2%) appeared to facilitate more adhesion of LGG compared with the mucus isolated from <6-month-old infants, albeit the difference was not statistically significant (43.3%, *p* = 0.26; Figure 2). In contrast, Bb12 showed significantly higher adherence to mucus isolated from <6-month-old infants (27.8%) when compared with mucus from >6-month-old infants (37.3%; *p* = 0.05).

**Figure 3:**
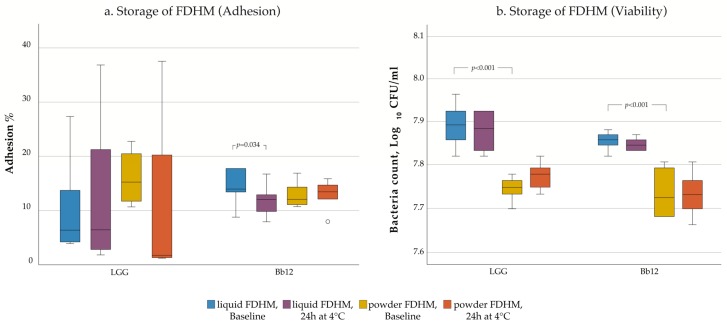
Mucus adhesion (**a**) and stability (**b**) of *L. rhamnosus* GG and *B. lactis* Bb12 in fortified donor human milk (FDHM) after storage at 4 °C for 24 h are presented with box plots. Outliers are marked with a circle (o).

Finally, storage did not significantly affect probiotic adhesion to intestinal mucus (Figure 3a). Interestingly, only Bb12 in liquid FDHM saw a notable decline in its adhesion capacity after storage for 24 h at 4 °C (Baseline: 14.3%, Storage: 11.9%; *p* = 0.034). When assessed by the traditional culturing method, the viability of LGG and Bb12 was maintained even after 24 h at 4 °C as there were no statistically significant decreases in the number of viable cells of the probiotic preparations (*p* > 0.05, Figure 3b). However, there was a significant difference in the CFU/mL of both bacteria between liquid and powder FDHM (LGG: 7.9 log_10_ CFU/mL (liquid FDHM), 7.7 log_10_ CFU/mL (powder FDHM), *p* < 0.001; Bb12: 7.8 log_10_ CFU/mL (liquid FDHM), 7.7 log_10_ CFU/mL (powder FDHM), *p* < 0.001).

## 4. Discussion

### 4.1. Strain Dependence of the Adhesion Properties

The results obtained from the present work are in agreement with previous reports [13,15]. Current findings show that both LGG and Bb12 adhered well to our mucus model. This can be partly explained by the fact that both strains are acid-resistant [16,17]. According to Collado and coworkers, acid-resistant bifidobacteria show better adhesive properties when compared with their acid-sensitive derivative [18]. Moreover, genomic analysis indicates that LGG presents cell wall-bound pili which are composed of proteins that have been shown to promote mucus adhesion. The SpaABC (Spa for sortase-mediated pilus assembly) pilus is composed of three pilin subunits: SpaA, SpaC and SpaB. More specifically, each pilus tip and backbone structure consists of SpaC pilin that is essential for the mucus binding of LGG [19]. Therefore, it can be speculated that the increased adhesion noted in our study might be partly due to the presence of pili. However, Sybesma and colleagues have noted that LGG isolated from dairy products was lacking the gene encoding for SpaC pilin compared with the American Type Culture Collection (ATCC) deposited LGG [20]. Thus, the cultivation method that is used for the large-scale production of probiotics is an important element influencing their health properties. There is increasing evidence that bifidobacteria also encode and express pili on their cell surface. Regarding Bb12, this bacterium harbors three proteins that are homologous to proteins documented to participate in sortase-dependent pili formation [21]. Additionally, two more extracellular proteins were isolated which are homologous to proteins reported to bind to human mucus. We also observed that when LGG and Bb12 were tested directly on infant mucus, Bb12 adhered well (28%–37%) but less compared with LGG (43%–49%). This could be attributed to the origin of the probiotic strains; LGG was originally isolated from the healthy human intestine, whereas Bb12 originates from Chr. Hansen’s collection of dairy cultures. When LGG and Bb12 were used in combination, no interference in adhesion was noted but instead, in line with previous studies [22], the adhesion of LGG appeared to be slightly, albeit statistically insignificantly, increased. The same was observed for Bb12 in the presence of LGG.

### 4.2. Role of DHM on the Adhesion of Probiotics

Human milk is a food matrix with a complex composition containing a myriad of different nutrients, bacterial and human cells, and immune-modulating components, as well as human milk oligosaccharides. However, it is not yet known whether DHM can serve as an optimal carrier for specific probiotics as it can play a critical role in their entrapment and thus interfere with their adhesion to the infant intestinal mucus. Research indicates that LGG can adhere to the milk fat globule membrane (MFGM) and decrease its adhesion to intestinal cells [23]. Interestingly, we found that probiotics suspended in buffer, adhered significantly better compared to DHM. This could be explained by two possible mechanisms: either a component of the milk is interacting with mucin glycoproteins and/or with the probiotics. Κ-casein, a protein present in human milk, has a C-terminal whose sequence is similar to that of mucin [24]. The concentration of this protein is increased after Holder pasteurization of human milk and could potentially prevent the attachment of bacteria to the mucus glycoproteins [25]. Mannose-binding lectins (MBL) are proteins also detected in human milk whose binding activity is not affected by Holder pasteurization [26,27]. Despite being a minor constituent, mannose moieties can be found in the structure of MUC2, the major colonic mucin in humans [28]. Depending on the glycosylation and degradation degrees of the intestinal mucus, this could indicate that human milk MBLs with high affinity for MUC2 mannose receptors could potentially bind to the isolated mucus and interfere with the probiotic adhesion. In addition to that, mucin 1, which is thermally stable after pasteurization and rich in mannose structures, has been identified as one of the major mucins of human milk [25,29,30]. A recent analysis of the LGG genome has revealed two lectin-like proteins, Llp1 and Llp2, with high binding specificity for D-mannose and mannan [31]. There is thus a possibility that LGG attaches to mucin 1 or other mannose-containing human milk glycoproteins through Llp1 and Llp2. Both LGG and Bb12 consist of a Gram-positive cell wall rich in peptidoglycans [21,32]. Soluble toll-like receptor 2 (sTLR2), shown to be present in high concentration in human milk, is a molecule responsible for recognizing bacterial peptidoglycan that could associate with both probiotics and prevent their mucosal adhesion [33].

In general, the best adhesion of LGG was achieved when it was suspended in buffer rather than when milk was present. Recently, it was reported that donor human milk can be safely stored up to 8 months after Holder pasteurization, without compromising its macronutrient and energy content [34]. One component of DHM that might contribute to this finding is human milk macrophages, the survival of which is decreased to a small extent by pasteurization temperature [35,36,37]. Indeed, a recent study found that the SpaCBA pili of LGG are key molecules for adhering to macrophages [38]. Similarly, analysis of the extracellular proteome of Bb12 revealed that two identified proteins are involved in the binding of manganese, an essential trace element that is also present in human milk [21]. Although pasteurization has a negative impact on the concentration/activity of some bioactive constituents of human milk, it does not affect the content of human milk oligosaccharides (HMO) [39]. Growth in the presence of a combination of human milk oligosaccharides (3′sialyllactose and 6′sialyllactose) was shown to substantially increase the adhesion of *Bifidobacterium longum* subsp. *infantis* to epithelial cells. However, in contrast to this bacterium, neither LGG or Bb12 are able to grow in the presence of HMOs [40]. On the other hand, these compounds are structurally very similar to mucus glycoproteins as they are both recognized by the same outer-membrane enzyme systems [41]. In addition, LGG, contrary to Bb12, has been shown to ferment L-fucose, which is a major constituent of both mucus and HMOs [40,42,43]. HMOs could, therefore, act as decoy receptors for probiotics, interfering with their adhesion to the mucus layer.

Polyunsaturated fatty acids (PUFA) are known for their antimicrobial properties and have also been reported in human milk. Kankaanpää and colleagues showed that 40 μg/mL of different PUFA (α-linolenic acid, arachidonic acid, and docosahexaenoic acid) suppress the mucus adhesion of LGG but not its viability [44]. Supplementary to that, daily milk intakes (around 750 mL/day in the first 4 months of life) contain an average concentration of 157, 134, and 66 μg/mL of α-linolenic acid, arachidonic acid, and docosahexaenoic acid, respectively [45,46]. These concentrations are higher than the PUFA concentration tested by Kankaanpää and coworkers, indicating the possible involvement of these molecules in the mucosal adhesion of probiotics.

Human milk is also rich in antimicrobial proteins and proteolytic enzymes including lysozyme, defensins, and cathelicidins [47,48]. Lysozyme, an enzyme that degrades bacterial cell wall peptidoglycans, has been reported to be decreased after Holder pasteurization, however, 60%−76% of its activity is still retained [49,50,51]. In addition, Ouwehand and coworkers showed a marked decrease in the mucus binding of LGG after the probiotic was pretreated with lysozyme [52]. Nevertheless, lysozyme did not have any effect on the adhesion capacity of Bb12. Regarding defensins, we are not sure if they affect the probiotic adhesion as there are contradicting results in the literature [53,54,55]. Cathelicidins, on the other hand, may play a dual role regarding adhesion as they could act on both mucus and probiotics. For instance, cathelicidin LL-37 was found to bind both mucin and negatively charged molecules on the cell wall of Gram-positive bacteria [56,57]. In our study, probiotics were subjected to centrifugation before their supplementation to DHM. Specifically, this step could damage the extracellular polymeric substance (EPS) layer of LGG, unmasking its pili that are responsible for its adhesion to intestinal mucus [58,59]. At the same time, stripping the EPS from the probiotics before their supplementation to DHM could have negative effects on the probiotic viability. To support this, Lebeer and colleagues showed that the viability of an EPS knock-out LGG mutant was compromised in the presence of LL-37 [54]. However, there are no available data on the effect of LL-37 on Bb12.

### 4.3. Age Dependency of the Adhesion Ability of Probiotics to Intestinal Mucus

In accordance with previous reports, we have found that the adhesion of the Bb12 to mucus from infants younger than 6 months of age was significantly better compared with that of infants more than 6 months of age. More specifically, Arboleya and coworkers confirmed that Bb12 adhered significantly better to mucus from 2-month-old infants than to that obtained from 6-month-old infants [60]. It has been noted that not all probiotic strains can adhere in a similar capacity to mucus from different age groups [13,61]. This indicates that the age of the individual might be an important factor when determining the adhesion properties and intended use of probiotics. However, the two mucus isolates used in the current study were not characterized. Thus, the reported probiotic adhesion difference between the two mucus could be due to various reasons. Firstly, differences may exist in the mucin oligosaccharide composition and the glycosylation patterns. For example, mucus isolated from < 6-month infants may be richer in mucin oligosaccharides that are utilized from the tested probiotics and favor their adhesion compared with >6-month infant mucus [62]. Secondly, the difference in the adhesion could be explained by a possible degradation of mucus isolated from 6–12-month infants. According to Midtvedt and colleagues, breastfed infants less than 4 months of age showed a delayed mucus degradation compared with mucus from infants aged 6–9 months old that demonstrated increased degradation of mucin [63]. In this case, it can be hypothesized that diet is one variable that influences the mucus degradation. Interestingly, another possible explanation could be that DHM contains glycoproteins such as lactoferrin which might bind the studied probiotics, thus leaving the intestinal mucus intact.

### 4.4. Influence of Storage

Despite the lack of standardization, two major in vitro models are used to assess probiotic adhesion: the intestinal mucus model and the cell line model. The results between these two methods vary widely [64]. In the current study, we chose to use the intestinal mucus model due to the fact that the mucus layer is the first interphase for host–probiotic interaction in the gastrointestinal tract. Previous reports have shown that subject age should be taken into consideration when determining probiotic adhesion properties—another reason why we found the mucus model more appropriate than the cell line model. The model has its limitations. Laparra and Sanz have reported that the mucus model cannot properly distinguish hydrophobic binding interactions from mucus binding interactions, making interpretation of results challenging. In addition, isolated mucus alone does not appropriately describe the in vivo situation and, therefore, a combination of both mucus and cell culture models might be appropriate for future work. This could provide more information as it would include both host and mucus interactions. Finally, another drawback of the current method is the use of a radioactive-based assay that has led to safety, waste disposal, and cost concerns. Vesterlund and coworkers have shown, however, that radioactive labels in adhesion assays are superior to fluorescence tagging or staining, especially in the case of bacteria with poor adhesion properties [65].

The results obtained from the adhesion experiment showed that the adhesion capacity of probiotics after their supplementation in FDHM was not affected by the subsequent storage at 4 °C for 24 h, nor by the type of fortifier used. Supplementary to that, when the same samples were cultured, the probiotic viability was maintained after storage and was not affected by the liquid or powdered FDHM. This is also in accordance with a previous study conducted by our group which showed that the viability of these specific probiotics was maintained in DHM for 24 h at 4 °C [66]. In NICUs, fortified donor milk can be stored at 4 °C for up to 24 h, making the results clinically relevant for preterm infants in similar units. However, these data may not be extrapolated to preterm infants. Lastly, we found differences in the CFU/mL of both probiotics between liquid and powdered FDHM that could be due to differences in their composition and final osmolality. The two HMFs tested might have different carbohydrate content or different carbohydrate origin. Therefore, the HMF polysaccharides might be broken down by the probiotics to a different degree depending on the type of fortifier.

### 4.5. Limitations

The results from the present study indicate that supplementation of specific probiotics in DHM or FDHM may change their ability to adhere to intestinal mucus glycoproteins. However, the picture may be different when assessed in vivo. Indeed, one great limitation of the current study was the omission of the in vitro digestion. Based on previous studies, passage through the stomach and small intestine could have a negative impact on the viability and the in vitro adhesive capacity of LGG and Bb12 to intestinal mucus [52,67]. However, as discussed above, human milk may influence the probiotics by either protecting them from digestion or interfering with their adhesion. Therefore, future studies should focus on investigating how the adhesion of probiotics is affected by the digestion of probiotic-supplemented human milk. The nutrient composition of donor milk was not characterized before or after Holder pasteurization so we are unable to point out which of the milk components had the strongest influence in the adhesion of the tested probiotics.

Another limitation of the current study was the absence of mucus from preterm infants. We experienced difficulties in finding preterm mucus and for this reason, we decided to include mucus from infants of < 6 months and 6–12 months. Thus, the current results cannot be extrapolated for preterm infants. Besides, we did not determine the composition of the two mucus isolates, we cannot be certain whether the differences in the adhesion between the two age groups are due to distinct glycosylation patterns or degradation of the mucus.

## 5. Conclusions

Our results suggest that the presence of donor human milk but not fortification with human milk fortifiers is an important element that determines the efficacy of probiotic adhesion to intestinal mucus. This factor along with specific probiotics should be considered when developing future infant feeding regimens. However, caution should be used when extrapolating these results to the in vivo situation. The current in vitro model did not include a digestion step, which possibly affects the probiotic adhesion. As the current study evaluated the mucus adhesion capacity of specific probiotic strains affected by two specific human milk fortifiers at a specific dose and for certain age groups, it is understandable that our observations may not be extrapolated for other probiotic strains or different age groups. Therefore, the effect of human milk fortifiers on probiotic adhesion properties should always be assessed on a case-to-case basis. Finally, given the increased adhesion of probiotics suspended in buffer, other matrices should be further studied.

## Supplementary Materials

The following are available online at https://www.mdpi.com/2072-6643/12/1/182/s1, Figure S1: Mucus adhesion of *L. rhamnosus* GG (1) and *B. lactis* Bb12 (2) in fortified donor human milk (FDHM) or after initial mucus treatment with FDHM as presented with box plot with outliers marked with a circle (o).

## References

[1] United Nations Inter-Agency Group for Child Mortality Estimation (UN IGME) (2019). Levels & Trends in Child Mortality: Report 2019. Estimates Developed by the United Nations Inter-Agency Group for Child Mortality Estimation.

[2] Sullivan M.C., Msall M.E., Miller R.J. (2012). 17-year outcome of preterm infants with diverse neonatal morbidities: Part 1, impact on physical, neurological, and psychological health status. J. Spec. Pediatr. Nurs..

[3] Carroll K., Herrmann K.R. (2013). The cost of using donor human milk in the NICU to achieve exclusively human milk feeding through 32 weeks postmenstrual age. Breastfeed. Med..

[4] Gephart S.M., Newnam K.M. (2019). Closing the gap between recommended and actual human milk use for fragile infants: What will it take to overcome disparities?. Clin. Perinatol..

[5] Ziegler E.E. (2001). Breast-milk fortification. Acta Paediatr..

[6] Brown J.V., Embleton N.D., Harding J.E., McGuire W. (2016). Multi-nutrient fortification of human milk for preterm infants. Cochrane Database Syst. Rev..

[7] Agostoni C., Buonocore G., Carnielli V.P., De Curtis M., Darmaun D., Decsi T., Domellöf M., Embleton N.D., Fusch C., Genzel-Boroviczeny O. (2010). Enteral nutrient supply for preterm infants: Commentary from the European Society of Paediatric Gastroenterology, Hepatology and Nutrition Committee on Nutrition. J. Pediatr. Gastroenterol. Nutr..

[8] Sharpe J., Way M., Koorts P.J., Davies M.W. (2018). The availability of probiotics and donor human milk is associated with improved survival in very preterm infants. World J. Pediatr..

[9] Hill C., Guarner F., Reid G., Gibson G.R., Merenstein D.J., Pot B., Morelli L., Canani R.B., Flint H.J., Salminen S. (2014). Expert consensus document: The International Scientific Association for Probiotics and Prebiotics consensus statement on the scope and appropriate use of the term probiotic. Nat. Rev. Gastroenterol. Hepatol..

[10] Monteagudo-Mera A., Rastall R.A., Gibson G.R., Charalampopoulos D., Chatzifragkou A. (2019). Adhesion mechanisms mediated by probiotics and prebiotics and their potential impact on human health. Appl. Microbiol. Biotechnol..

[11] Collado M.C., Cernada M., Baüerl C., Vento M., Pérez-Martínez G. (2012). Microbial ecology and host-microbiota interactions during early life stages. Gut Microbes.

[12] Bin-Nun A., Bromiker R., Wilschanski M., Kaplan M., Rudensky B., Caplan M., Hammerman C. (2005). Oral probiotics prevent necrotizing enterocolitis in very low birth weight neonates. J. Pediatr..

[13] Kirjavainen P.V., Ouwehand A.C., Isolauri E., Salminen S.J. (1998). The ability of probiotic bacteria to bind to human intestinal mucus. FEMS Microbiol. Lett..

[14] Ouwehand A.C., Kirjavainen P.V., Grönlund M.-M., Isolauri E., Salminen S.J. (1999). Adhesion of probiotic micro-organisms to intestinal mucus. Int. Dairy J..

[15] Juntunen M., Kirjavainen P.V., Ouwehand A.C., Salminen S.J., Isolauri E. (2001). Adherence of probiotic bacteria to human intestinal mucus in healthy infants and during rotavirus infection. Clin. Diagn. Lab. Immunol..

[16] Gorbach S.L., Goldin B.R. (1989). Lactobacillus Strains and Methods of Selection. U.S. Patent.

[17] Li Q., Chen Q., Ruan H., Zhu D., He G. (2010). Isolation and characterisation of an oxygen, acid and bile resistant *Bifidobacterium* animalis subsp. lactis Qq08. J. Sci. Food Agric..

[18] Collado M.C., Gueimonde M., Sanz Y., Salminen S. (2006). Adhesion properties and competitive pathogen exclusion ability of bifidobacteria with acquired acid resistance. J. Food Prot..

[19] Kankainen M., Paulin L., Tynkkynen S., von Ossowski I., Reunanen J., Partanen P., Satokari R., Vesterlund S., Hendrickx A.P.A., Lebeer S. (2009). Comparative genomic analysis of *Lactobacillus rhamnosus* GG reveals pili containing a human-mucus binding protein. Proc. Natl. Acad. Sci. USA.

[20] Sybesma W., Molenaar D., van IJcken W., Venema K., Kort R. (2013). Genome instability in *Lactobacillus rhamnosus* GG. Appl. Environ. Microbiol..

[21] Gilad O., Svensson B., Viborg A.H., Stuer-Lauridsen B., Jacobsen S. (2011). The extracellular proteome of *Bifidobacterium* animalis subsp. lactis BB-12 reveals proteins with putative roles in probiotic effects. Proteomics.

[22] Ouwehand A.C., Isolauri E., Kirjavainen P.V., Ölkkö S.T., Salminen S.J. (2000). The mucus binding of *Bifidobacterium lactis* Bb12 is enhanced in the presence of *Lactobacillus* GG and *Lact. delbrueckii* subsp. *bulgaricus*. Lett. Appl. Microbiol..

[23] Guerin J., Burgain J., Francius G., El-Kirat-Chatel S., Beaussart A., Scher J., Gaiani C. (2018). Adhesion of *Lactobacillus rhamnosus* GG surface biomolecules to milk proteins. Food Hydrocoll..

[24] Thorn D.C., Ecroyd H., Carver J.A., Holt C. (2015). Casein structures in the context of unfolded proteins. Int. Dairy J..

[25] Ma Y., Zhang L., Wu Y., Zhou P. (2019). Changes in milk fat globule membrane proteome after pasteurization in human, bovine and caprine species. Food Chem..

[26] Trégoat V., Montagne P., Béné M.-C., Faure G. (2002). Changes in the mannan binding lectin (MBL) concentration in human milk during lactation. J. Clin. Lab. Anal..

[27] Cossey V., Jeurissen A., Bossuyt X., Schuermans A. (2009). Effect of pasteurisation on the mannose-binding lectin activity and the concentration of soluble CD14 in human milk. J. Hosp. Infect..

[28] Johansson M.E.V., Larsson J.M.H., Hansson G.C. (2011). The two mucus layers of colon are organized by the MUC2 mucin, whereas the outer layer is a legislator of host–microbial interactions. Proc. Natl. Acad. Sci. USA.

[29] Parry S., Hanisch F.G., Leir S.-H., Sutton-Smith M., Morris H.R., Dell A., Harris A. (2006). N-Glycosylation of the MUC1 mucin in epithelial cells and secretions. Glycobiology.

[30] Liu B., Newburg D.S. (2013). Human milk glycoproteins protect infants against human pathogens. Breastfeed. Med..

[31] Petrova M.I., Imholz N.C.E., Verhoeven T.L.A., Balzarini J., Damme E.J.M.V., Schols D., Vanderleyden J., Lebeer S. (2016). Lectin-Like Molecules of *Lactobacillus rhamnosus* GG Inhibit Pathogenic *Escherichia coli* and *Salmonella* Biofilm Formation. PLoS ONE.

[32] Lahtinen S.J., Haskard C.A., Ouwehand A.C., Salminen S.J., Ahokas J.T. (2004). Binding of aflatoxin B1 to cell wall components of *Lactobacillus rhamnosus* strain GG. Food Addit. Contam..

[33] LeBouder E., Rey-Nores J.E., Rushmere N.K., Grigorov M., Lawn S.D., Affolter M., Griffin G.E., Ferrara P., Schiffrin E.J., Morgan B.P. (2003). Soluble forms of Toll-like receptor (TLR)2 capable of modulating TLR2 signaling are present in human plasma and breast milk. J. Immunol..

[34] De Waard M., Mank E., van Dijk K., Schoonderwoerd A., van Goudoever J.B. (2018). Holder-pasteurized human donor milk: How long can it be preserved?. J. Pediatr. Gastroenterol. Nutr..

[35] Cress C., Paxson C.L. (1977). Breast milk macrophages. Pediatr. Res..

[36] Gibbs J.H., Fisher C., Bhattacharya S., Goddard P., Baum J.D. (1977). Drip breast milk: Its composition, collection and pasteurization. Early Hum. Dev..

[37] Björkstén B., Burman L.G., De Château P., Fredrikzon B., Gothefors L., Hernell O. (1980). Collecting and banking human milk: To heat or not to heat?. Br. Med. J..

[38] Vargas García C.E., Petrova M., Claes I.J.J., De Boeck I., Verhoeven T.L.A., Dilissen E., von Ossowski I., Palva A., Bullens D.M., Vanderleyden J. (2015). Piliation of *Lactobacillus rhamnosus* GG Promotes Adhesion, Phagocytosis, and Cytokine Modulation in Macrophages. Appl. Environ. Microbiol..

[39] Bertino E., Coppa G.V., Giuliani F., Coscia A., Gabrielli O., Sabatino G., Sgarrella M., Testa T., Zampini L., Fabris C. (2008). Effects of holder pasteurization on human milk oligosaccharides. Int. J. Immunopathol. Pharmacol..

[40] Thongaram T., Hoeflinger J.L., Chow J., Miller M.J. (2017). Human milk oligosaccharide consumption by probiotic and human-associated bifidobacteria and lactobacilli. J. Dairy Sci..

[41] Marcobal A., Barboza M., Sonnenburg E.D., Pudlo N., Martens E.C., Desai P., Lebrilla C.B., Weimer B.C., Mills D.A., German J.B. (2011). *Bacteroides* in the infant gut consume milk oligosaccharides via mucus-utilization pathways. Cell Host Microbe.

[42] Becerra J.E., Yebra M.J., Monedero V. (2015). An l-fucose operon in the probiotic *Lactobacillus rhamnosus* GG is involved in adaptation to gastrointestinal conditions. Appl. Environ. Microbiol..

[43] Arnold J.W., Simpson J.B., Roach J., Bruno-Barcena J.M., Azcarate-Peril M.A. (2018). Prebiotics for lactose intolerance: Variability in galacto-oligosaccharide utilization by intestinal *Lactobacillus rhamnosus*. Nutrients.

[44] Kankaanpää P.E., Salminen S.J., Isolauri E., Lee Y.K. (2001). The influence of polyunsaturated fatty acids on probiotic growth and adhesion. FEMS Microbiol. Lett..

[45] Institute of Medicine and the Committee on Nutritional Status during Pregnancy and Lactation (1991). Milk Volume. Nutrition during Lactation.

[46] Koletzko B. (2016). Human milk lipids. Ann. Nutr. Metab..

[47] Murakami M., Dorschner R.A., Stern L.J., Lin K.H., Gallo R.L. (2005). Expression and secretion of cathelicidin antimicrobial peptides in murine mammary glands and human milk. Pediatr. Res..

[48] Baricelli J., Rocafull M.A., Vázquez D., Bastidas B., Báez-Ramirez E., Thomas L.E. (2015). β-defensin-2 in breast milk displays a broad antimicrobial activity against pathogenic bacteria. J. Pediatr..

[49] Evans T.J., Ryley H.C., Neale L.M., Dodge J.A., Lewarne V.M. (1978). Effect of storage and heat on antimicrobial proteins in human milk. Arch. Dis. Child..

[50] Wills M.E., Han V.E., Harris D.A., Baum J.D. (1982). Short-time low-temperature pasteurisation of human milk. Early Hum. Dev..

[51] Viazis S., Farkas B.E., Allen J.C. (2007). Effects of high-pressure processing on immunoglobulin A and lysozyme activity in human milk. J. Hum. Lact..

[52] Ouwehand A.C., Tölkkö S., Salminen S. (2001). The effect of digestive enzymes on the adhesion of probiotic bacteria in vitro. J. Food Sci..

[53] De Keersmaecker S.C.J., Braeken K., Verhoeven T.L.A., Perea Vélez M., Lebeer S., Vanderleyden J., Hols P. (2006). Flow cytometric testing of green fluorescent protein-tagged *Lactobacillus rhamnosus* GG for response to defensins. Appl. Environ. Microbiol..

[54] Lebeer S., Claes I.J.J., Verhoeven T.L.A., Vanderleyden J., Keersmaecker S.C.J.D. (2011). Exopolysaccharides of *Lactobacillus rhamnosus* GG form a protective shield against innate immune factors in the intestine. Microb. Biotechnol..

[55] Wang X.-F., Tian F., Cao R.-M., Li J., Wu S.-M., Guo X.-K., Chen T.-X. (2015). Antimicrobial activity of human β-defensins against lactic acid bacteria. Nat. Prod. Res..

[56] Felgentreff K., Beisswenger C., Griese M., Gulder T., Bringmann G., Bals R. (2006). The antimicrobial peptide cathelicidin interacts with airway mucus. Peptides.

[57] Kainulainen V., Loimaranta V., Pekkala A., Edelman S., Antikainen J., Kylväjä R., Laaksonen M., Laakkonen L., Finne J., Korhonen T.K. (2012). Glutamine synthetase and glucose-6-phosphate isomerase are adhesive moonlighting proteins of *Lactobacillus crispatus* released by epithelial cathelicidin LL-37. J. Bacteriol..

[58] Bell C.H., Camesano T.A. The Effects of Centrifugation and Filtration as Pre-Treatments in Bacterial Retention Studies. https://www.jyi.org/2005-june/2005/6/8/the-effects-of-centrifugation-and-filtration-as-pre-treatments-in-bacterial-retention-studies.

[59] Lebeer S., Verhoeven T.L.A., Francius G., Schoofs G., Lambrichts I., Dufrêne Y., Vanderleyden J., De Keersmaecker S.C.J. (2009). Identification of a gene cluster for the biosynthesis of a long, galactose-rich exopolysaccharide in *Lactobacillus rhamnosus* GG and functional analysis of the priming glycosyltransferase. Appl. Environ. Microbiol..

[60] Arboleya S., Ruas-Madiedo P., Margolles A., Solís G., Salminen S., de los Reyes-Gavilán C.G., Gueimonde M. (2011). Characterization and in vitro properties of potentially probiotic *Bifidobacterium* strains isolated from breast-milk. Int. J. Food Microbiol..

[61] Ouwehand A.C., Isolauri E., Kirjavainen P.V., Salminen S.J. (1999). Adhesion of four *Bifidobacterium* strains to human intestinal mucus from subjects in different age groups. FEMS Microbiol. Lett..

[62] Tassell M.L.V., Miller M.J. (2011). Lactobacillus adhesion to mucus. Nutrients.

[63] Midtvedt A.-C., Carlstedt-Duke B., Midtvedt T. (1994). Establishment of a mucin-degrading intestinal microflora during the first two years of human life. J. Pediatr. Gastroenterol. Nutr..

[64] Laparra J.M., Sanz Y. (2009). Comparison of in vitro models to study bacterial adhesion to the intestinal epithelium. Lett. Appl. Microbiol..

[65] Vesterlund S., Paltta J., Karp M., Ouwehand A.C. (2005). Measurement of bacterial adhesion—In vitro evaluation of different methods. J. Microbiol. Methods.

[66] Mantziari A., Aakko J., Kumar H., Tölkkö S., du Toit E., Salminen S., Isolauri E., Rautava S. (2017). The impact of storage conditions on the stability of *Lactobacillus rhamnosus* GG and *Bifidobacterium* animalis subsp. lactis Bb12 in human milk. Breastfeed. Med..

[67] Miettinen M., Alander M., von Wright A., Vuopio-Varkila J., Marteau P. (1999). The survival of and cytokine induction by lactic acid bacteria after passage through a gastrointestinal model. Microb. Ecol. Health Dis..

